# CRISPR-Cas9 Mediated NOX4 Knockout Inhibits Cell Proliferation and Invasion in HeLa Cells

**DOI:** 10.1371/journal.pone.0170327

**Published:** 2017-01-18

**Authors:** Naser Jafari, Hyunju Kim, Rackhyun Park, Liqing Li, Minsu Jang, Andrew J. Morris, Junsoo Park, Cai Huang

**Affiliations:** 1 Markey Cancer Center, University of Kentucky, Lexington, Kentucky, United States of America; 2 Division of Biological Science and Technology, Yonsei University, Wonju, Republic of Korea; 3 Division of Cardiovascular Medicine, Department of Internal Medicine, University of Kentucky, Lexington, Kentucky, United States of America; University of Tasmania, AUSTRALIA

## Abstract

Increased expression of NOX4 protein is associated with cancer progression and metastasis but the role of NOX4 in cell proliferation and invasion is not fully understood. We generated NOX4 knockout HeLa cell lines using the CRISPR-Cas9 gene editing system to explore the cellular functions of NOX4. After transfection of CRISPR-Cas9 construct, we performed T7 endonuclease 1 assays and DNA sequencing to generate and identify insertion and deletion of the NOX4 locus. We confirmed the knockout of NOX4 by Western blotting. NOX4 knockout cell lines showed reduced cell proliferation with an increase of sub-G1 cell population and the decrease of S/G2/M population. Moreover, NOX4 deficiency resulted in a dramatic decrease in invadopodium formation and the invasive activity. In addition, NOX4 deficiency also caused a decrease in focal adhesions and cell migration in HeLa cells. These results suggest that NOX4 is required for both efficient proliferation and invasion of HeLa cells.

## Introduction

Reactive oxygen species (ROS) are involved in a diverse range of biological processes functioning as intracellular signaling molecules. NADPH oxidases are responsible for the generation of reactive oxygen species [1, 2]. NADPH oxidase 4 (NOX4) is a member of NOX/DUOX family, which includes 7 isoforms (NOX1-5 and DUOX 1–2) and NOX4 is one of the most abundant and extensively expressed NOX isoforms [3–5]. NOX4 is responsible for the production of H_2_O_2_, and the expression of NOX4 is particularly high in the kidney [6, 7]. Therefore many previous studies focused on the role of NOX4 in renal physiology and pathophysiology leading to the conclusion that NOX4 is a major source of ROS in diabetic nephropathy [8–10].

The role of NOX4 in cancer is also significant. Previous studies showed that NOX4 is upregulated in many cancers such as glioma, melanoma and thyroid cancer [11–13]. NOX4 is also reported to contribute to progression and metastasis in various cancers [14–16]. However, mechanisms by which NOX4 regulates cancer cell proliferation, survival and migration are not fully understood. Several reports indicate that NOX4 promotes cell proliferation by regulating cell cycle and by inhibiting apoptosis [12, 17, 18]. On the other hand, other studies indicate that NOX4 inhibits cell proliferation and also promotes apoptosis [19, 20]. While most of these studies used the small interfering RNA mediated knockdown system, genomic inactivation of NOX4 has not been reported in cancer cells. In this report, we generated NOX4 knockout HeLa cell lines using the CRISPR-Cas9 gene editing system, and we demonstrated that the NOX4 knockout attenuated cell proliferation and also reduced cell invasion. These results support the concept that NOX4 is required for the efficient cell proliferation and cell invasion of HeLa cells.

## Material and Methods

### Cell culture, cell cycle analysis and cell proliferation assay

HeLa human cervical cancer cells were from the American Type Culture Collection and were maintained in DMEM medium (Mediatech, Inc.) containing 10% fetal bovine serum (FBS), penicillin (100 U/ml) and streptomycin (100 μg/ml). HeLa cells were transfected with Safectine RU50 (SydLabs, Natick, MA) according to the manufacturer's protocol. For cell cycle analysis, cells were washed with phosphate buffered saline (PBS) and fixed with 70% ethanol. After centrifugation, cells were washed and resuspended in PBS containing 0.25 mg/ml propidium iodide (PI) and 10 mg/ml RNase A (Sigma, St. Louis, MO, USA). We used MTT assay to measure the cell proliferation. MTT [Thiazolyl Blue Tetrazolium Bromide] (CAS Number: 298-93-1) was purchased from RPI corp (Mt. Prospect, Il). Cells were suspended in DMEM containing 10% FBS, seeded in 96-well plates, and then incubated for an overnight. Next day, wells’ medium was replaced with 100 μL of FBS free medium containing 250 μg/ml MTT and incubated for 4 extra hours. Then supernatants were removed and 200 μL DMSO was added to each well. Plates were shaken for 10 min and the absorbance was measured at 570 nm by Cytation 5 Cell Imaging Multi-Mode Reader (BioTek, Winooski, VT).

### Generation of Knockout cell line with CRISPR/Cas9

Guide RNA sequences for CRISPR/Cas9 were designed at CRISPR design web site (http://crispr.mit.edu/), provided by the Feng Zhang Lab [21]. Insert oligonucleotides for human NOX4 gRNA #1 and #2 are 5’-CACCGGCACATGGGTAAAAGGATG-3’ / 5’-AAACTATCCTTTTACCCATGTGCC-3’ and 5’-CACCGACACTCTTGGCTTACCTCCG-3’ / 5’-AAACCGGAGGTAAGCCAAGAGTGTC-3’, respectively. The NOX4 guide RNA targets the exon 3 of NOX4 gene. The complementary oligonucleotides for guide RNAs (gRNAs) were annealed, and cloned into pX459 CRISPR/Cas9-Puro vector (Addgene, Cambridge, MA). HeLa cells were transfected with either pX459/gRNA #1 or pX459/gRNA #2 using Safectine RU50, according to the manufacturer's instructions. Two days after transfection, cells were treated with 1 μg/ml of puromycin for three days. After two weeks, colonies were isolated with the cloning cylinders, and the NOX4 sequences were analyzed with T7 endonuclease (T7E1) assay, DNA sequencing and Western blot. The antibody for NOX4 was purchased from ProSci (Poway, CA).

### Western blotting

The cells were harvested and lysed in RIPA buffer (50mM Tris-HCl pH 7.5, 150mM NaCl, 1% IPEGAL, 0.5% deoxycholate, 5mM EDTA) containing a protease inhibitor cocktail (Roche, Mannheim, Germany). For protein immunoblot analysis, polypeptides in whole cell lysates were resolved by SDS-PAGE and transferred to nitrocellulose membranes. The images were acquired using Odyssey (Licor bioscience, Lincoln, NE). The antibodies for NOX1 (Cat #:7921), NOX2 (Cat #:7923), NOX3 (Cat #:7925) and NOX4 (Cat #:7927) were from ProSci (Poway, CA). The antibodies for Caspase 3 (Cat #:9668), Cyclin D1 (Cat #: 2978), S6 Ribosomal Protein antibody (5G10), phospho S6 ribosomal protein [pSer235/236] (D57.2.2E) were from Cell Signaling Technology (Beverly, MA). The antibodies for Akt1 antibody (Y89), phospho-Akt [pSer473] antibody (EP2109Y) were from Abcam (Cambridge, MA). The antibody for NOX5 from Boster Biological Technology (Pleasanton, CA), the antibody for DUOX1 from GeneTex (Irvine, CA), the antibody for DUOX2 from Novus Biologicals (Littleton, CO), and the antibody for p22phox from Santa Cruz Biotechnology (San Diego, CA).

### T7 endonuclease (T7E1) assay

Genomic DNA was extracted from the cells and the genomic sequences containing the target sequences of NOX4 gRNA’s were PCR amplified using 5’-CAATGGGGTAGGGGCAGAAC-3’ forward primer, and 5’-TGCCTGAGGTTTCTTGTGTG-3’ reverse primer. Next, the nested PCR was conducted with 5’-TGGCAGTGCAGAACAGAAAGA-3’ forward primer, and 5’-AGCATAGTGCTGGAAATCCC-3’ reverse primer. PCR products were denatured and reannealed for heteroduplex formation. Finally 10 μl of reaction product was incubated with 2.5 unit of T7 Endonuclease in 37°C for 30 min, and the digested products were analyzed with the agarose gel electrophoresis.

### Real time quantitative PCR

We used quantitative PCR to measure the expression level of NOX4 and p22phox. Primers for NOX4 are 5’-CTG TGT CCT GGA GGA GCT GG-3’ (forward) and 5’-AAG CCA AGA GTG TTC GGC AC-3’ (reverse). p22phox primer sequences are 5’-GAA GTA CAT GAC CGC CGT GG-3’ (forward) and 5’-TGC TTG ATG GTG CCT CCG AT-3’ (reverse). Actin was used as a housekeeping gene to normalize Ct values. Sequences for Actin primer set are 5’-CAA CCG CGA GAA GAT GAC-3’ (forward) and 5’-AGG AAG GCT GGA AGA GTG-3’ (reverse). Primers were synthesized by Sigma Life Science. Total RNA of the cells was extracted by using PureLink RNA Mini Kit (Cat #:12183025) from Life Technologies Corporation. First-strand cDNA was synthesized from total purified RNA (0.5 to 1.0 μg) by using SuperScript^®^ III First-Strand Synthesis System (Cat #: 18080–051) from Life Technologies Corporation, according to the manufacturer's instructions. Quantitative reverse transcriptase PCR (RT-PCR) reactions were performed using SYBR Green PCR master mix reagents (Cat #: K0223) on an ABI Onestep Plus Real-Time PCR System (Applied Biosystems, Foster city, CA). The relative expression of each amplicon was analyzed by the ΔCt method.

### Measurement of H_2_O_2_ released from cells

100 μL of reaction mixture contain 50 μM Amplex (Cayman Chemicals, Ann Arbor, MI) and 0.1 U/ml horse radish peroxidase in Krebs-Ringer buffer (KRB, containing 135 mM NaCl, 5 mM KCl, 1 mM MgSO_4_, 1 mM CaCl_2_, 0.4 mM K_2_HPO_4_, 5.5 mM Glucose, 20 mM HEPES, pH 7.4) were added into each well of a 96-well plate and warmed at 37°C for 10 min. Cells were trypsinized, washed and re-suspended in with KRB at 2.5 × 10^6^ cells/ml. Cell suspensions (20 μL) were mixed with reaction mixtures in each well and incubated at 37°C for 10 min. 20 L of KRB without cells were added to the reaction mixtures as blank controls. Fluorescence was measured by using Cytation 5 Cell Imaging Reader (BioTek, Winooski, VT), with excitation 571 nm and emission 585 nm.

### Invasion assays and wound healing assay

Invasion assays were performed as described [22–24]. Briefly, one hundred microliters of Matrigel (1:30 dilution in serum-free DMEM medium) was added to each Transwell polycarbonate filter (6 mm diameter, 8 μm pore size, Corning) and incubated with the filters at 37°C for 4 hours. HeLa cells were trypsinized and washed three times with DMEM containing 1% FBS. The cells were resuspended in DMEM containing 1% FBS at a density of 1×10^6^ cells/ml. The cell suspensions (100 μl) were seeded into the upper chambers, and 600 μl of DMEM medium containing 20 ng/ml EGF were added to the lower chambers. The cells were allowed to invade for 24 hours (or as indicated) in a CO_2_ incubator, fixed, stained and quantitated. We utilized a wound healing assay to measure the cell migration. Control and NOX4 knockout cells were maintained in 35 mm dishes. When the dish was confluent with the cells, the cell layer was scratched by a 200 μl pipette tip, and cultured at 37°C for 24 h or 48 h. The cell image was captured and the average extent of wound closure was quantified by ImageJ software.

### Focal adhesion assays

Cells were plated on glass coverslips that were precoated with fibronectin. For immunofluorescence staining, the cells were fixed with 4% paraformaldehyde, permeabilized with 0.5% Triton X-100, blocked with 5% BSA in PBS, and then incubated with anti-paxillin antibody and anti-zyxin antibody. Paxillin and zyxin were then visualized by incubating with DyLight 549 Conjugated goat Anti-mouse IgG (H+L) and DyLight 488 Conjugated goat Anti-Rabbit IgG (H+L). Paxillin and zyxin staining were viewed by using a Nikon Eclipse Ti TIRF microscope equipped with a 60x, 1.45 NA objective, CoolSNAP HQ2 CCD camera (Roper Scientific). Images were acquired and analyzed by using NIS-Elements (Nikon, Tokyo, Japan). To quantify the number and area of focal adhesions, paxillin and zyxin immunofluorescence images were thresholded to include only focal adhesions and the number and area were calculated by using the software. Paxillin antibody was purchased from Millipore (Bedford, MA), and zyxin antibody was from Epitomics (Burlingame, CA).

### Invadopodia assay

Glass-bottom dishes were coated with 100 μl of warm Cy3-conjugated gelatin (0.2 mg/ml) in PBS containing 2% sucrose. The coated dishes were dried, fixed with pre-chilled glutaraldehyde solution (0.5% in PBS), washed with PBS and then reduced with 5 mg/ml of sodium borohydride in PBS. The dishes were washed extensively with PBS and then incubated with DMEM containing 10% FBS and antibiotics for 1h. Cells were seeded and incubated on the gelatin surface for 24 h in a CO_2_ incubator. After 24 hours, cells were fixed and immunostained with anti-cortactin antibody. Cortactin antibody was purchased from Cell Signaling (Beverly, MA).

## Results

### Generation of NOX4 knockout cell lines with CRISPR-Cas9 gene editing system

**Fig 1:**
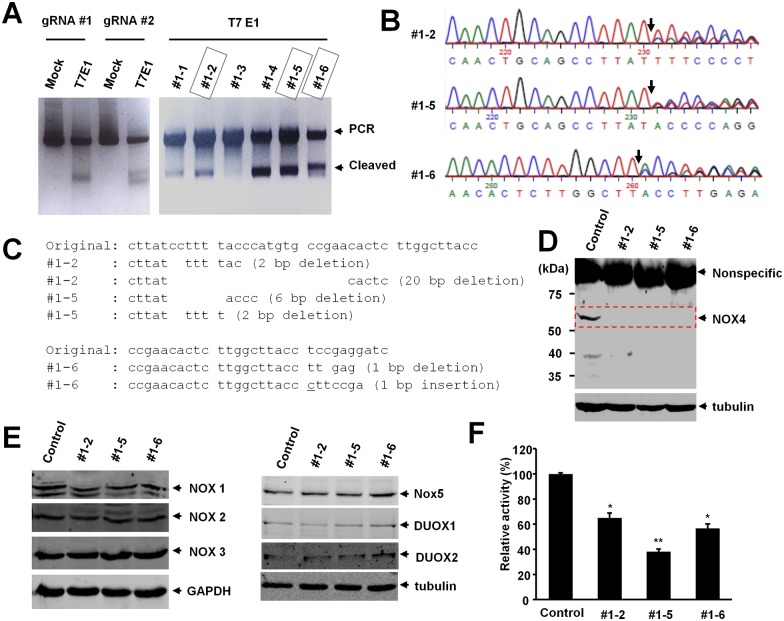
Generation and validation of NOX4 knockout (KO) HeLa cell lines. (**A**) T7 endonuclease 1(T7E1) assay. HeLa cells were transiently transfected with either pX459/NOX4 gRNA #1 or pX459/NOX4 gRNA #2, and genomic PCR products were analyzed by T7E1 assay (left panel). HeLa cells transfected with pX459/NOX4 gRNA’s were selected with puromycin and single colonies were isolated. The genomic PCR product of each clone was analyzed by T7E1 assay (right panel). Experiments were repeated three times with similar observations, and representative images are shown. (**B**) DNA sequence analysis showed the presence of the NOX4 mutation in clones #1–2, #1–5 and #1–6. The black arrows indicate the heterogeneous genomic DNA sequences in each cell lines. (**C**) CRISPR-Cas9 introduced the insertion and deletion (indel) mutation in the target sites. (**D**) Western blot of NOX4 knockout cell lines. Equal amount of HeLa control and NOX4 knockout cell lysates were probed with anti-NOX4 antibody. Experiments were repeated three times with similar observations, and representative data is shown. (**E**) NOX4 knockout did not influence NOX1, NOX2, NOX3, NOX5, DUOX1 and DUOX2 levels. Protein levels of NOX1, NOX2, NOX3, NOX5, DUOX1 and DUOX2 in parental HeLa cells and three clones of NOX4 knockout cells. (**F**) NOX4 knockout showed lower H_2_O_2_ production. The levels of H_2_O_2_ were measured with the Amplex Red assay in triplicate. The graph shows the average and the standard deviation (SD). Control vs knockout cells. *: P <0.005, **: P <0.0001.

We used the CRISPR-Cas9 gene editing system to generate NOX4 knockout cell lines. The oligonucleotides for the guide RNA were designed, synthesized and cloned into pX459 vector, and we evaluated the gene editing activity of each guide RNA with the transient transfection of either pX459/NOX4 gRNA #1 (gRNA #1) or pX459/NOX4 gRNA #2 (gRNA #2) followed by T7 endonuclease 1 (T7E1) assays. The cleaved bands by T7E1 enzyme indicate that insertion or deletion mutations (indel) were introduced into the genomes (Fig 1A, left panel). Next, we isolated single colonies from the transfected cells and analyzed the indel mutations in these isolated cell lines (Fig 1A, right panel). Then we analyzed the nucleotide sequences of the PCR products of target DNA, and confirmed the indel mutations were introduced into the genome (Fig 1B and 1C). Finally, Western blot with anti-NOX4 antibody showed that the expression of NOX4 protein was abolished in NOX4 knockout cell lines (Fig 1D). These results indicate that NOX4 knockout cell lines were generated by CRISPR-Cas9 system.

Because the CRISPR-Cas9 mediated knockout system produces indel mutation, the level of NOX4 mRNA was not significantly reduced in NOX4 knockout cells (S1A Fig). We also examined the expression of NOX1, NOX2, NOX3, NOX5, DUOX1 and DUOX2 by Western blot, and we did not find any significant change in their expression (Fig 1E). In addition, we also examined the expression of p22phox, the binding partner of NOX4. Although quantitative RT-PCR analysis revealed that the expression of p22phox mRNA was slightly increased in NOX4 knockout cells, the expression of p22phox protein was not significantly changed in NOX4 knockout cells (S1B, S1C and S1D Fig).

The main function of NOX4 is the production of ROS such as H_2_O_2_. Therefore we examined whether NOX4 knockout cells showed a reduction in the level of ROS with the Amplex Red assay, a fluorescent assay to detect H_2_O_2_. NOX4 knockout cells showed significantly reduced ROS production in comparison to control cells (Fig 1F). These results indicate that NOX4 depletion inhibits ROS generation.

### NOX4 deficient cells exhibit decreased proliferation and viability

**Fig 2:**
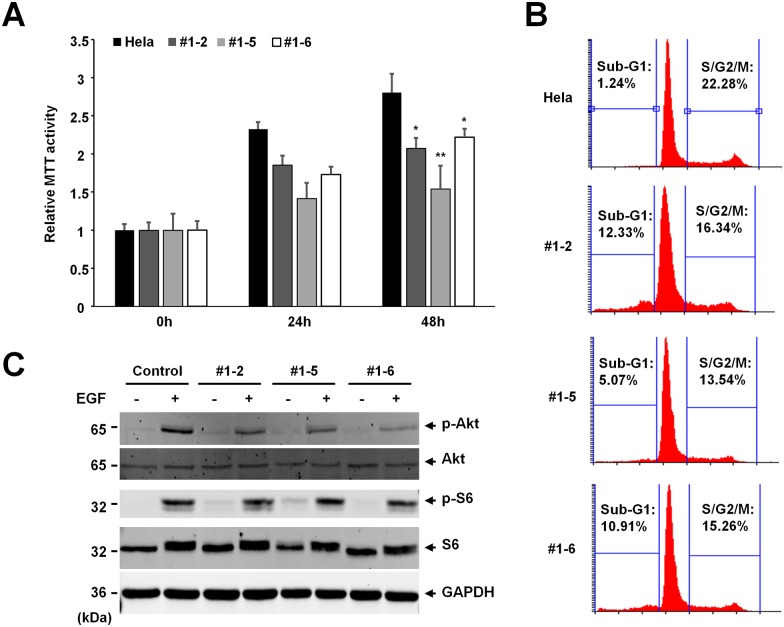
NOX4 knockout inhibits cell proliferation. (**A**) Proliferation of three NOX4 knockout cell lines was quantitated by MTT assay. HeLa control and knockout clones were seeded into 24 well plate and the MTT assay were performed in quadruplicate. The graph shows the average and the standard deviation (SD). Control vs knockout cells. *: P <0.005, **: P <0.00005. (**B**) NOX4 knockout HeLa cells were analyzed with flow cytometry. The percentage of cells at sub-G1 and S/G2/M was measured, and is shown in the graph. Experiment was repeated three times with similar observations, a representative data is shown. (**C**) NOX4 knockout may affect activation of Akt phosphorylation. NOX4 depleted cells showed significantly lower levels of phospho-Akt in comparison to parental normal cell. However, NOX4 knockout did not affect phosphorylation of S6 Ribosomal Protein. Cells were serum starved by incubation in DMEM containing 1% FBS for 10 hrs, then exposed to EGF (20ng/ml) for 20 min before collection of cells for analysis. Experiments were repeated three times with similar observations, and representative results are shown.

When we generated the NOX4 knockout cell lines, we observed a reduction in cell proliferation in NOX4 knockout cells. For this reason, we examined the proliferation of NOX4 knockout cells. HeLa control and NOX4 knockout cells were seeded into 24 well plates, and we performed MTT assay to examine the cell proliferation. The proliferation of three NOX4 knockout cell lines was significantly reduced compared to HeLa control cells (Fig 2A). To confirm the role of NOX4 in cell proliferation, we used flow cytometry to analyze the cell cycle in NOX4 knockout cells. Compared with HeLa control cells, the NOX4 knockout HeLa cells showed increased population of cells in the sub G1 phase, suggesting a higher rate of apoptosis in the NOX4 knockout cells (Fig 2B). In addition, the cell populations in S/G2/M in NOX4 knockout cells were reduced, indicating that NOX4 deficiency also attenuates cell growth (Fig 2B). Together the proliferation assay and cell cycle analysis indicate that NOX4 is required for the efficient cell growth in HeLa cells.

Recent reports indicate that NOX4 regulates cancer cell proliferation by regulating the PI3K pathway [14]. Therefore we examined whether NOX4 knockout affects the downstream of PI3K signaling. We examined the level of phospho-Akt upon EGF treatment. While the baseline levels of Akt in all NOX4 knockout cells is equal to the parental control cells, the level of phospho-Akt in NOX4 knockout cells showed the lower level of phosphor-Akt upon EGF treatment (Fig 2C). However the level of phosphor-S6 was not affected by NOX4 knockout. We also examined the expression levels of cyclin D1 protein and caspase 3, but we did not detect any significant change in NOX4 knockout cells (S2 Fig). These data suggest that NOX4 regulate PI3K signaling.

### NOX4 deficient cells exhibit decreased migration and invasion

**Fig 3:**
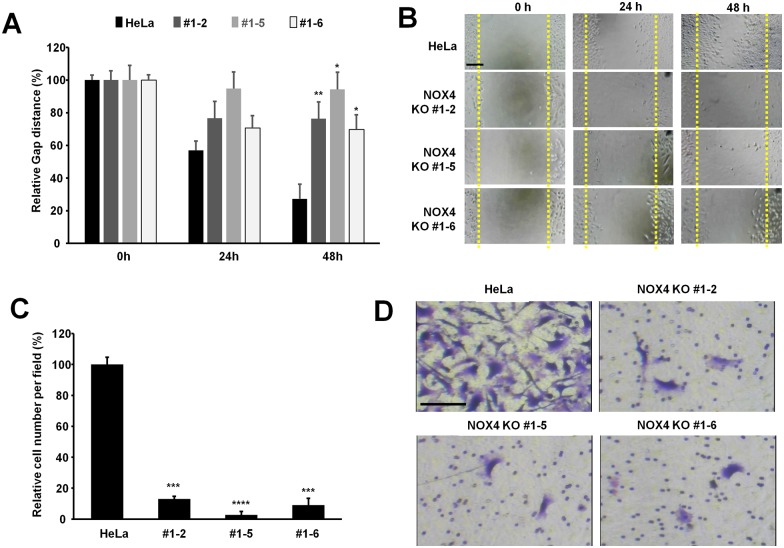
NOX4 knockout inhibits cell migration and invasion. (**A, B**) NOX4 knockout inhibits the cell migration of HeLa cells. Equal numbers of cells were plated onto a 6 well plate, and cells were scraped. After 24 h and 48 h, the wound gaps were photographed using an inverted microscope and quantified using imageJ software. Bars: 100 μm. The graph shows the mean and the standard deviation (SD). Control vs NOX4 knockout cells. *: P <0.0001, **: P <0.00001, n = 6. (**C, D**) NOX4 knockout inhibits the cell invasion. Invasion of control and NOX4 knockout cells through matrigel in response to EGF (20 ng/ml for 24 h) was assessed by the invasion assay. Bars: 100 μm. The graph shows the average and the standard deviation (SD). Control vs NOX4 knockout cells. ***: P <0.005, ****: P <0.0005, n = 20. Experiments were repeated three times with similar observations, and representative images of the invasive cells are shown.

Next, we examined whether NOX4 knockout affects the cell invasion and migration. First, we examined the cell migration of NOX4 knockout cells using the wound healing assay. Equal numbers of cells were plated, and the cell monolayer was scraped and monitored for wound closure. After 24 h and 48 h, the wound gaps were quantified for statistical analysis. While HeLa control cells migrated into the wound, the cell migration of NOX4 knockout cells was reduced significantly. These findings indicate that NOX4 deficiency reduces HeLa cell migration (Fig 3A and 3B).

Next, we examined the invasive activity of NOX4 knockout cells by using a matrigel invasion assay. We added EGF in the lower chamber and examined the cell invasion after 24 h. HeLa control cells showed the active cell invasion whereas the NOX4 knockout cells revealed significantly lower invasive activity (Fig 3C and 3D). These results collectively suggest that NOX4 is required for both cell migration and invasion.

### NOX4 deficient cells exhibit decreased focal adhesion and invadopodium formation

**Fig 4:**
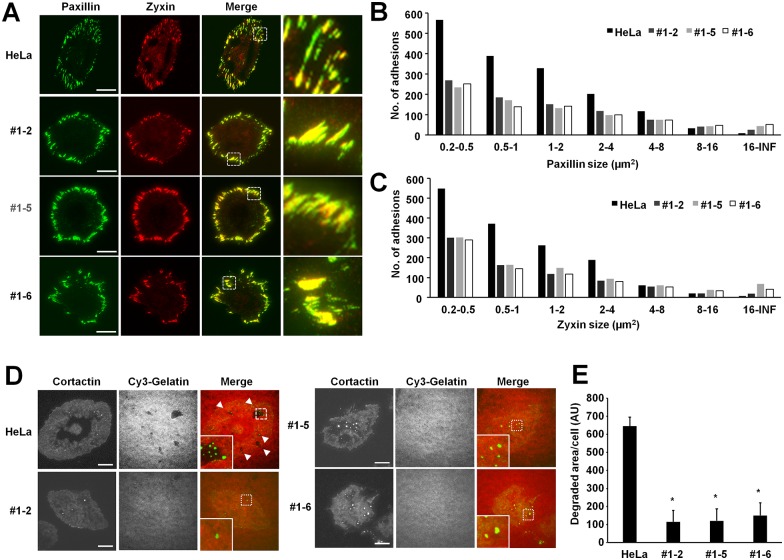
NOX4 knockout reduces focal adhesion and invadopodium formation in HeLa cells. (**A**) Cells were seeded in fibronectin coated glass-bottom dishes, and stained with anti-paxillin antibody (green) and anti-zyxin antibody (red). The images were analyzed with TIRF microscope. Bars: 20 μm. (**B, C**) The area distribution of focal adhesions in 20 cells were analyzed and shown in graph. (**D**) NOX4 is required for the efficient invadopodium formation. HeLa control and NOX4 knockout cells were cultured on coverslip coated with Cy3-gelatin (red) and stained with Cortactin (green). Gelatin degradation is visualized as darker areas. Arrowheads denote the gelatin degraded areas. Bars: 20 μm. (**E**) The degradation of gelatin was quantified using image analysis software. The graph shows the average and the standard error of the mean (SEM). HeLa control vs NOX4 knockout cells, *: P<0.05, n = 20.

Because NOX4 knockout cells showed reduced cell migration and cell invasion, we examined the focal adhesion formation in NOX4 knockout cells. HeLa control and NOX4 knockout cells were plated on glass bottom dishes precoated with fibronectin and stained with anti-paxillin antibody and anti-zyxin antibody (Fig 4A). NOX4 knockout cells had reduced number of focal adhesion but the sizes of their focal adhesions were larger. Moreover focal adhesions in these cells were lined up around of the edge of the cells (Fig 4A). Quantitative analysis indicated that the knockout NOX4 decreased focal adhesions up to 2 fold and the decrease was mainly accounted for by small focal adhesions (<4 μm^2^) (Fig 4B and 4C). We next examined invadopodium formation in the NOX4 knockout cells. HeLa control and NOX4 knockout cells were cultured in gelatin coated dishes and we measured gelatin degradation. HeLa control cells could degrade the gelatin, however NOX4 knockout cells exhibited a very low level of gelatin degradation (Fig 4D and 4E). These results suggest that NOX4 is required for the efficient focal adhesion and invadopodium formation in HeLa cells.

In addition, we analyzed the expression NOX4 and p22phox in cervical cancers using the database since we used HeLa cervical cancer cell line. Geo profiles database in NCBI showed that the expression of NOX4 and p22phox are significantly elevated in cervical cancers suggesting NOX4-p22phox is activated in cervical cancer for migration and invasion (S3 Fig).

## Discussion

CRISPR-Cas9 gene editing system is very useful in cancer cell biology because it provides a way to generate gene knockouts in human cancer cell lines. In this report, we generated NOX4 knockout HeLa cell lines by using CRISPR-Cas9 gene editing system. As far as we are aware, this is the first report to study the NOX4 knockout in human cancer cells. Because our study did not employ transient transfection or small interfering RNA-mediated knockdown, this approach avoids potential side effects caused by the transfection reagent or the ectopic RNA expression.

Many reports have been published about the role of NOX4 in human cancers. However, the role of NOX4 in cell proliferation and apoptosis is not fully understood. While many reports suggest that NOX4 contributes to cell proliferation, several reports demonstrated that NOX4 inhibits cell proliferation and also contributes to apoptosis in hepatocytes [19, 25, 26]. Our results indicate that NOX4 is required for cell proliferation. Cell cycle analysis showed that NOX4 knockout HeLa cells had elevated level of sub G1 population and reduced level of S/G2/M population (Fig 2B). One interpretation of these observations is that NOX4 has cell type specific functions.

Accumulating evidences suggest that the elevated level of ROS promotes the proliferation and invasion of tumor cells [27, 28]. Because NOX4 expression contributes to the production of ROS, the inhibition of NOX4 expression by siRNA suppresses the proliferation or invasion of tumors cells as well as ROS formation [12, 29]. The production of ROS by the elevated expression of NOX4 contributes to the activation of PI3K signaling and FAK signaling [14, 30], which in turn regulate invadopodium formation and focal adhesion dynamics [31, 32]. Furthermore, NOX4 is shown to increase IL-6 and IL-8 production in renal cell carcinoma [33]. Therefore, the treatment of antioxidant such as vitamin E to cancer cells attenuates the proliferation and invasion of tumor cells by lowering ROS [12, 14]. In this report, we used HeLa cells to examine the role of NOX4 in the proliferation and invasion of tumor cells and previous studies showed the antioxidant treatment in HeLa cells inhibits the cell proliferation and invasion [34]. For this reason, the phenotype of NOX4 knockout HeLa cells is quite close to that of the antioxidant treatment.

Focal adhesions are sites of cell adhesion to extracellular matrix, and dynamic regulation of focal adhesions promote cancer cell migration and invasion [35]. NOX4 knockout HeLa cells showed reduced numbers of focal adhesions but the remaining focal adhesions were larger suggesting that the focal adhesions in the NOX4 knockout cells are less dynamic than in the parental HeLa cell line. Therefore the less dynamic focal adhesions in the NOX4 knockout cells likely contribute to the reduced cell migration and invasion that we observed. In addition, we also observed reduced gelatin degradation activity of the NOX4 knockout cells. Invadopodia are the docking sites of matrix metalloproteinase (MMP) with extracellular matrix, and the protease activity of MMP is crucial for the cell invasion [36]. For this reason, it is likely that reduced migration and invasion activity of the NOX4 knockout HeLa cells is a result of the reduced number of focal adhesions/deficiency in focal adhesion turnover and formation of invadopodia and their associated activities. Further studies will be needed to elucidate the regulatory mechanism by which NOX4 regulates focal adhesion and invadopodium formation.

## Supporting Information

S1 Fig Relative expression levels of NOX4 and p22phox. (A) Quantitative PCR was conducted to analyze the expression levels of NOX4 gene. Expression of NOX4 did not show significant difference in NOX4 knockout cells, n = 3. (B) p22phox mRNA expression levels were slightly increased in NOX4 knockout cells. Data are presented as mean ± SEM of 3 independent experiments. Control vs knockout cells, *: P<0.05, n = 3. (C, D) NOX4 knockout did not affect the protein levels of p22phox. Control vs knockout, P>0.05, n = 3.
(TIF)

S2 Fig NOX4 knockout did not affect the expression of cyclin D1 or caspase 3 cleavage. NOX4 knockout did not influence expression of cyclin D1. Moreover, depletion of NOX4 did not affect activation process of caspase 3.
(TIF)

S3 Fig Expression of NOX4 and p22phox are elevated in cervical cancers. Data on expression of NOX4 and p22phox in normal cervix and cervical cancer (GDS3233) were collected from GEO profiles (http://www.ncbi.nlm.nih.gov). Normal cervix vs Cervical cancer, *: P<0.05, **: P<0.01.
(TIF)
