# Supplementary figures and images for: CRISPR-Cas9 Mediated NOX4 Knockout Inhibits Cell Proliferation and Invasion in HeLa Cells
Source: PLoS One. 2017 Jan 18;12(1):e0170327. doi: 10.1371/journal.pone.0170327 (PMC5242459; doi:10.1371/journal.pone.0170327)

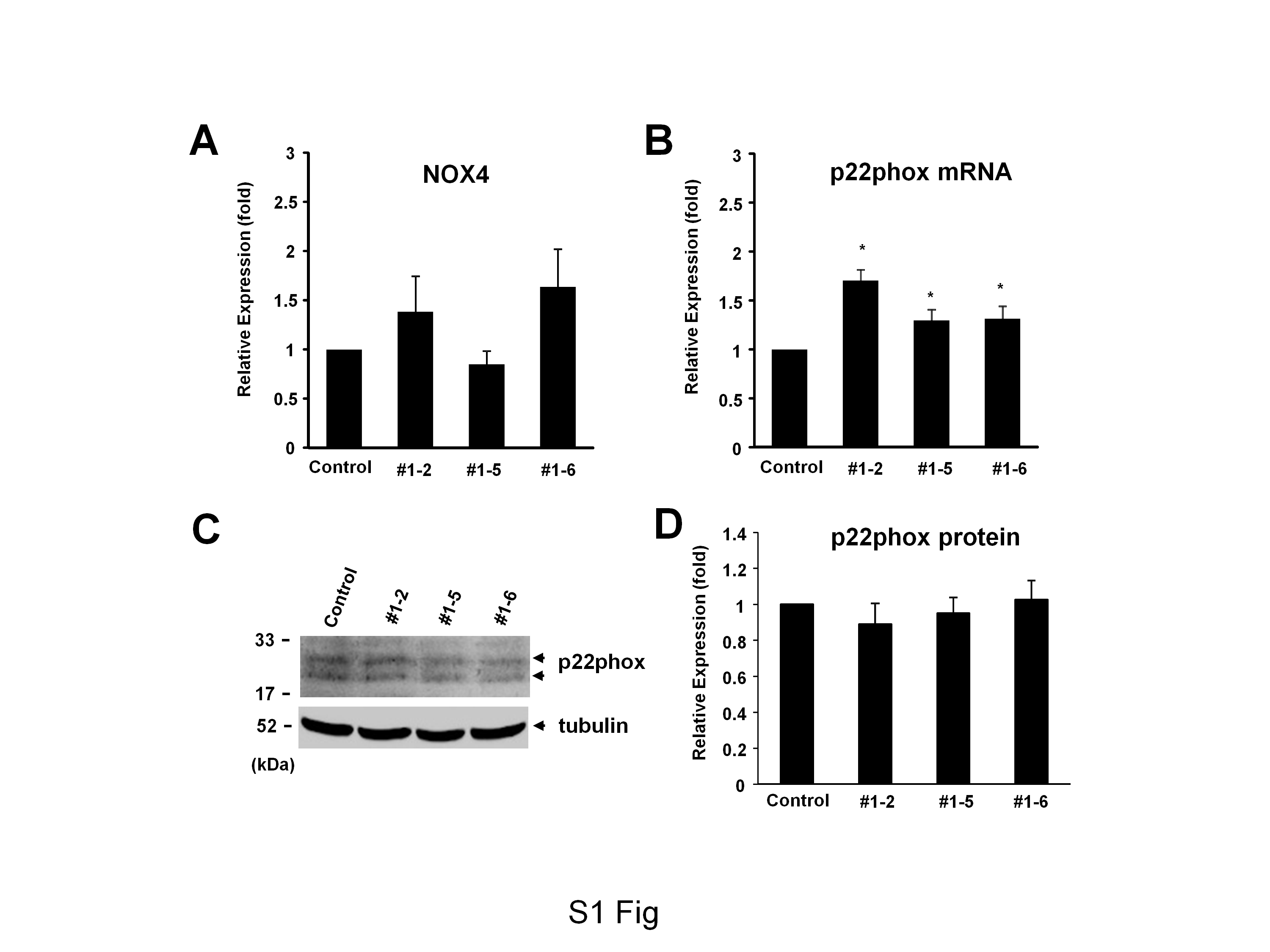

Supplement: S1 Fig — (A) Quantitative PCR was conducted to analyze the expression levels of NOX4 gene. Expression of NOX4 did not show significant difference in NOX4 knockout cells, n = 3. (B) p22phox mRNA expression levels were slightly increased in NOX4 knockout cells. Data are presented as mean ± SEM of 3 independent experiments. Control vs knockout cells, *: P<0.05, n = 3. (C, D) NOX4 knockout did not affect the protein levels of p22phox. Control vs knockout, P>0.05, n = 3. (TIF) [file pone.0170327.s001.TIF]

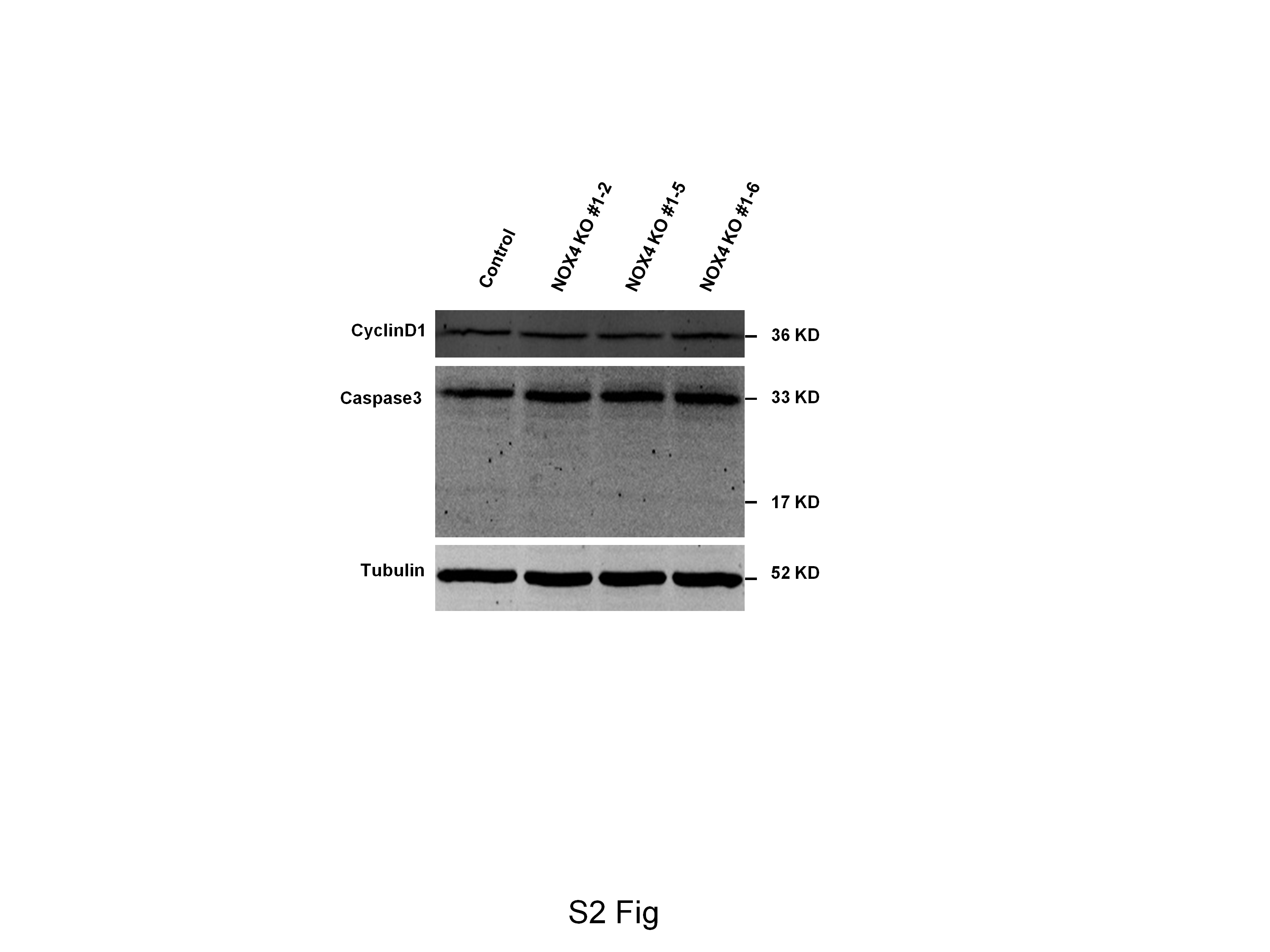

Supplement: S2 Fig — NOX4 knockout did not influence expression of cyclin D1. Moreover, depletion of NOX4 did not affect activation process of caspase 3. (TIF) [file pone.0170327.s002.TIF]

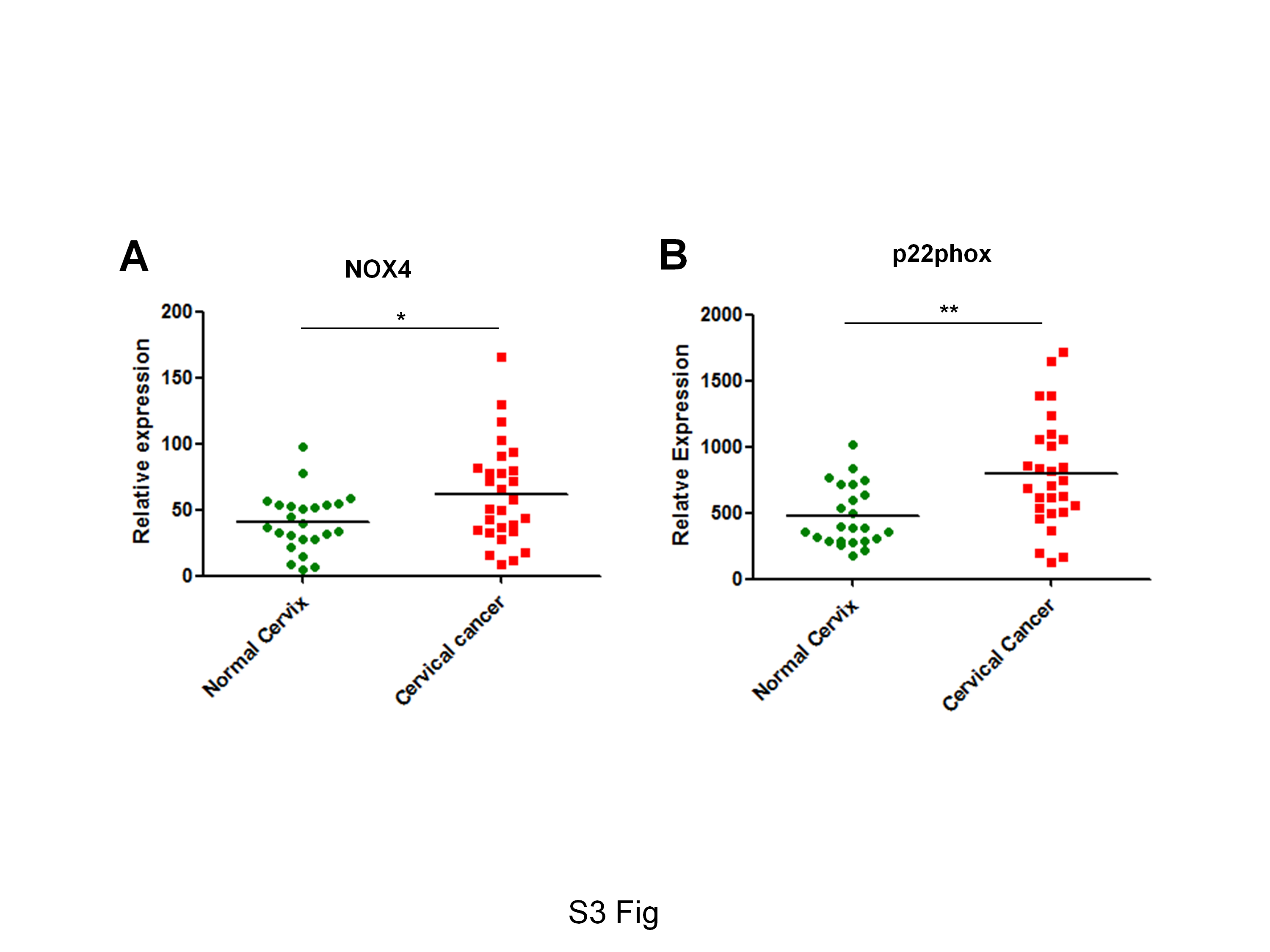

Supplement: S3 Fig — Data on expression of NOX4 and p22phox in normal cervix and cervical cancer (GDS3233) were collected from GEO profiles (http://www.ncbi.nlm.nih.gov). Normal cervix vs Cervical cancer, *: P<0.05, **: P<0.01. (TIF) [file pone.0170327.s003.TIF]
